# Pectoral muscle cross-sectional area correlates with bone mineral density in postmenopausal women

**DOI:** 10.3389/fmed.2026.1781151

**Published:** 2026-03-06

**Authors:** Min Xue, Xiao-Hui You, Xiong-Yi Wang, You-Jia Xu

**Affiliations:** 1 Department of Ultrasound, The Second Affiliated Hospital of Soochow University, Suzhou, Jiangsu, China; 2 Center for Medical Ultrasound, Suzhou Municipal Hospital, The Affiliated Suzhou Hospital of Nanjing Medical University, Gusu School, Nanjing Medical University, Suzhou, Jiangsu, China; 3 Department of Orthopaedics, The Second Affiliated Hospital of Soochow University, Suzhou, Jiangsu, China

**Keywords:** bone mineral density (BMD), chest computed tomography (CT), dual-energy X-ray absorptiometry (DXA), osteoporosis (OP), pectoral muscle area (PMA)

## Abstract

**Objective:**

To evaluate the correlation between pectoralis muscle and bone mineral density (BMD) in postmenopausal women with estrogen deficiency. Furthermore, the degree of bone loss can be initially assessed by chest muscle area.

**Methods:**

This is a retrospective study. 500 subjects were included in this study from August 2023 to August 2024. The participants were classified into normal, osteopenia, and osteoporosis groups. We analyzed the correlation between the age, BMI, PMA and BMD. The correlation between PMA and BMD was tested by multiple linear regression, after correction for age and BMI.

**Results:**

A total of 338 subjects were finally included in the study after exclusion criteria. There was good agreement between the two measurement workers (ICC = 0.980, *p* < 0.05). Age, BMI and PMA were strongly correlated with BMD. PMA was positively correlated with lowest BMD (*r* = 0.448). Multiple linear regression showed no multicollinearity between age, BMI and PMA. The formula was: Lowest BMD = 0.858 – 0.005 ^*^ age + 0.006 ^*^ BMI + 0.005 ^*^ PMA.

**Conclusions:**

Decreased muscle mass increases the risk of osteoporosis prevalence. Simple measurements from routine chest CT can provide information about BMD and offer a way to evaluate osteoporosis.

## 1 Introduction

Osteoporosis (OP) and sarcopenia are common age-related disorders that often coexist in older adults (1). OP is marked by reduced bone mass and deteriorated bone structure, leading to greater fragility and fracture risk (2). Sarcopenia involves the loss of muscle mass and function, contributing significantly to frailty and disability (3). Critically, their coexistence synergistically raises the risk of falls, fractures, and mortality (4).

Menopause represents a critical milestone in female aging and is accompanied by profound endocrine changes, most notably a decline in estrogen levels (5). Estrogen deficiency has been implicated as a key factor underlying the higher prevalence of both osteoporosis and sarcopenia observed in postmenopausal women compared with age-matched men (6). Beyond hormonal regulation, increasing evidence supports the concept that bone and skeletal muscle function as a closely integrated musculoskeletal unit (7). Consequently, alterations in muscle mass and function may have a direct impact on bone strength and mineral density, particularly in populations vulnerable to hormonal imbalance.

Although dual-energy X-ray bone densitometry (DXA) is the gold standard for measuring bone mineral density (BMD) and muscle mass throughout the body, it is not routinely performed in all clinical settings (8, 9). In contrast, computed tomography (CT) imaging is frequently employed for a variety of clinical indications, presenting an opportunity to assess musculoskeletal health without incurring additional radiation exposure or costs (10). Previous studies have demonstrated that skeletal muscle mass or cross-sectional area measured at various anatomical sites, such as the psoas muscle or thigh muscles, is associated with bone mineral density and osteoporosis risk in older adults (11). However, most of these studies relied on abdominal CT or site-specific muscle groups, which are not routinely acquired in general clinical practice, thereby limiting their applicability for opportunistic osteoporosis screening. Notably, the majority of existing studies have focused on mixed populations or male-dominated cohorts, while evidence specifically targeting postmenopausal women—who represent a high-risk group for both osteoporosis and sarcopenia—remains scarce. Recently, it has been suggested that pectoral muscle cross-sectional area at the T4 level in chest CT can assess the muscle condition of patients and has been proposed as a surrogate marker for sarcopenia (12). Chest CT is one of the most commonly performed imaging examinations in clinical practice, especially for health screening and pulmonary disease evaluation, making it a potentially valuable tool for opportunistic musculoskeletal assessment (13). However, despite growing interest in CT-derived muscle metrics, the relationship between pectoral muscle area (PMA) and bone mineral density in postmenopausal women has not been systematically investigated. Therefore, whether pectoral muscle area measured on routine chest CT can reflect bone mineral density in postmenopausal women remains unclear. Addressing this gap may provide a convenient and clinically applicable approach for opportunistic osteoporosis assessment.

In this context, the present study aimed to investigate the association between PMA and BMD in postmenopausal women, and to discuss the potential value of muscle measurements from chest CT scans as an opportunistic assessment of bone loss.

## 2 Methods

This retrospective study was approved by the Ethics Committee of The Second Affiliated Hospital of Soochow University (approval date: March 01, 2024; approval number: JD-HG-2024-018). Clinical data were retrospectively extracted from the electronic medical record system after ethical approval, and no additional contact with participants was required.

### 2.1 Study design and participants

**Figure 1:**
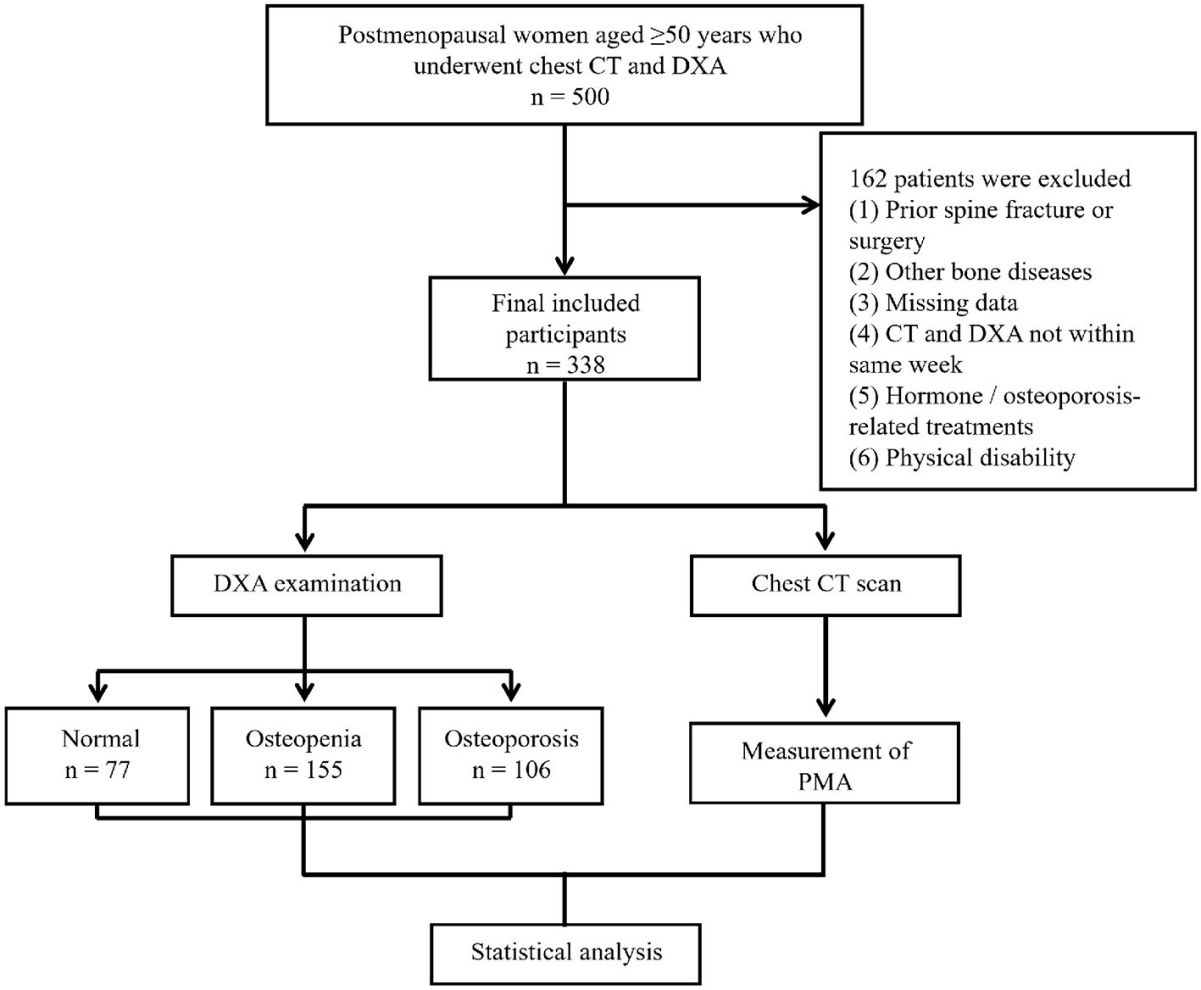
Flowchart of the study design and participant selection.

The flowchart of participant selection and study design is shown in Figure 1. This retrospective study included postmenopausal women aged over 50 years who had undergone both chest CT and DXA examinations at The Second Affiliated Hospital of Soochow University between August 2023 and August 2024. Participants were identified through the hospital's electronic medical records system. Exclusion criteria: (1) previous history of thoracolumbar spine fracture and surgery; (2) comorbid bony diseases such as spinal tuberculosis, bone tumor, ankylosing spondylitis, diffuse idiopathic osteomalacia, etc.; (3) patients with missing baseline data; (4) CT and DXA were not performed in the same week; (5) history of long-term use of corticosteroids or other hormonal treatments; (6) history of long-term calcium and vitamin D supplementation; (7) history of treatment with osteoporosis-related medications such as PTH and denosumab. 8. Individuals with physical disabilities due to other causes. A total of 338 study subjects were finally included. Patients' age, gender, body mass index (BMI) and other relevant baseline data were collected from medical records.

### 2.2 CT scanning parameters and image post-processing

**Figure 2:**
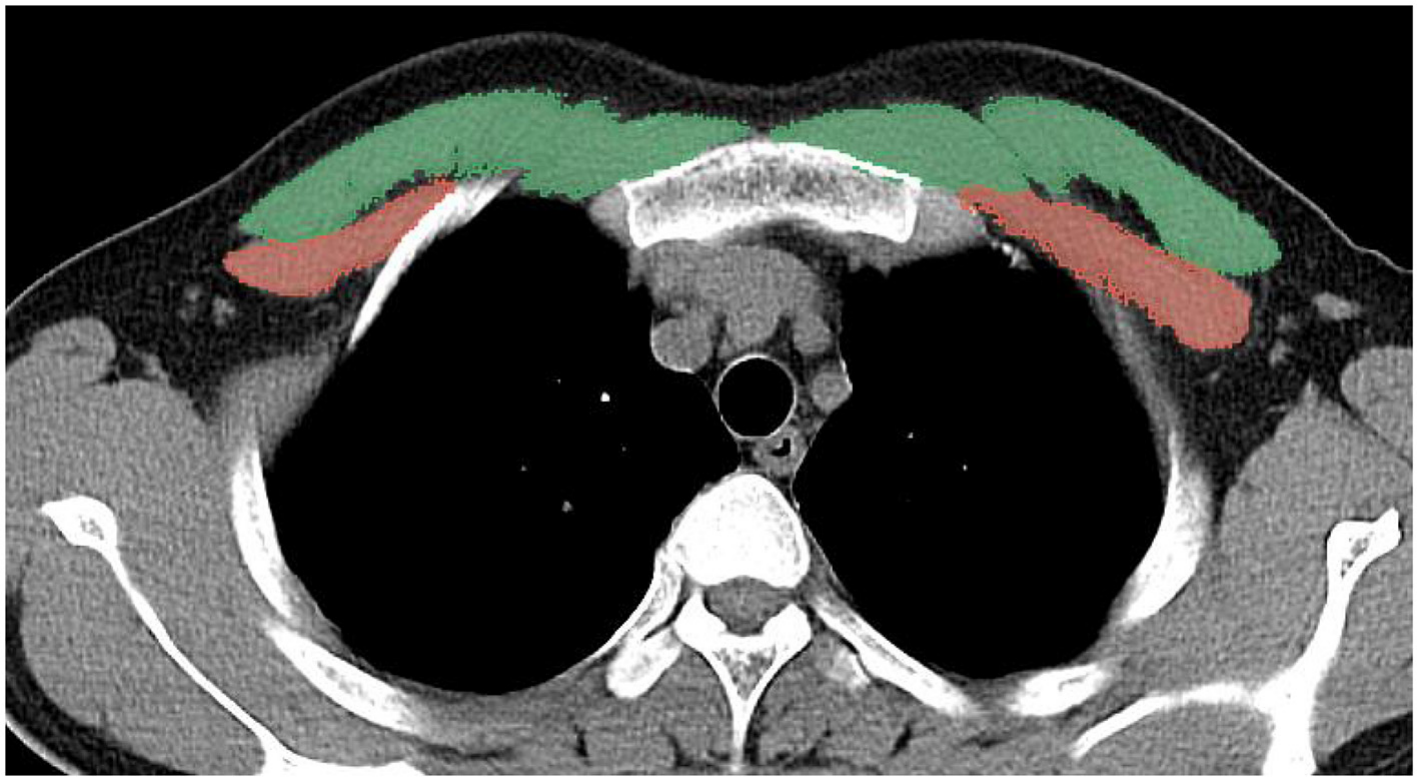
A method for determining PMA by CT horizontal plane of the T4 vertebrae, the pectoralis major is green and the pectoralis minor is red.

A 6-row spiral CT scanner (SOMATOM Emotion 6) from Siemens was used to perform multilayer routine chest CT examination of the study subjects. The spiral scanning area was in supine position and no contrast agent was used during the procedure. Chest CT imaging scanning parameters: tube voltage: 120 kv; tube current: automatic adjustment; layer thickness: 0.625–2 mm. Image analysis of CT was performed using the 3D Slicer. Image selection was performed at the level of the T4 vertebrae using the method described in this study (14). At the median level of the T4 vertebrae, the researcher manually drew the edges of the pectoralis major and pectoralis minor muscles, and the bilateral pectoralis muscle area (PMA) was automatically calculated by summing the pixel attenuation within a threshold range of −29 to +150 HU for skeletal muscle (Figure 2). All measurements were performed by two independent observers, who were unaware of the subject's DXA results, to avoid subjective influences on the measured data (15).

### 2.3 Bone mineral density examination

A GE Lunar Prodigy dual-energy X-ray bone mineral density meter was used to detect BMD in all study subjects. Measurements included BMD and *T* score of L1–4 and hip. BMD was expressed as g/cm^2^. According to the World Health Organization (WHO) diagnostic criteria, osteoporosis was defined as a *T*-score ≤ −2.5, osteopenia as a *T*-score between −1.0 and −2.5, and normal bone mass as a *T*-score ≥−1.0. Patient categorization in this article was based on the lowest *T* value in the DXA (16).

### 2.4 Statistical analysis

SPSS 26.0 was used for statistical analysis. The intraclass-correlation coefficient (ICC) was calculated to compare observer agreement. Inter-observer variability of muscle area measurements was determined using the method described by Bland and Altman, and consistency was determined by plotting the mean measurement difference of 1.96 standard deviation (SD) ±. Normally distributed variables are expressed as mean ± standard deviation, and non-normally distributed variables are expressed as median. Differences in continuous variables between multiple groups were tested using the ANOVA test (for consistency with normal distribution) and the Kruskal–Wallis test (for consistency with non-normal distribution), and Tukey's method was used for comparisons between any two groups. Differences in categorical data were tested using the chi-square test. Pearson correlation coefficients were used to analyze the correlation of each influential factor and scatter plots were drawn. *P* < 0.05 was considered statistically significant.

## 3 Results

### 3.1 Clinical baseline data

**Figure 3:**
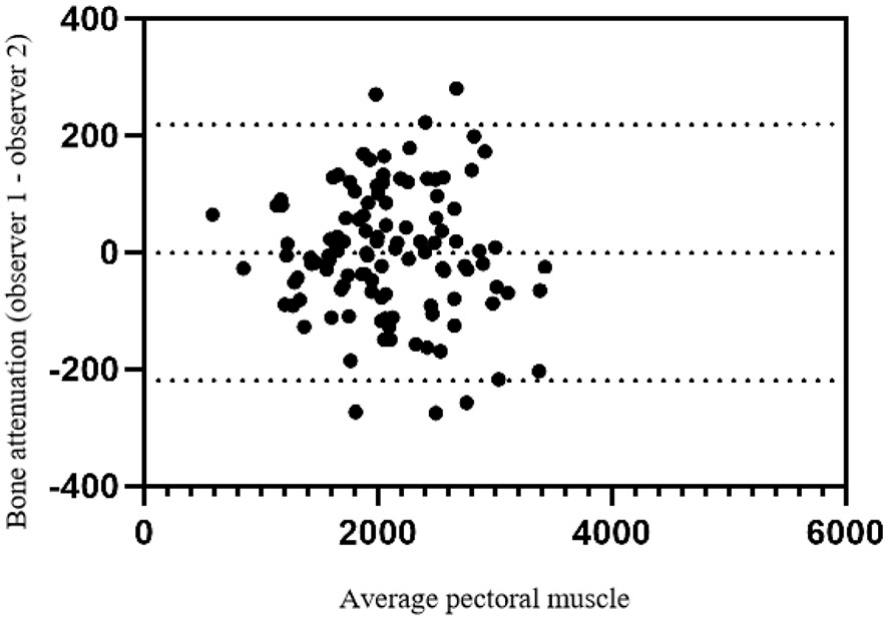
Bland Altman plots for the inter-observer of the pectoral muscle measurements on chest CT.

According to the inclusion criteria, 500 subjects were enrolled in this study. According to the exclusion criteria, 162 subjects were excluded. A total of 338 patients were finally included in the study. There was good agreement between the two measurement workers (ICC = 0.980, *p* < 0.05). Figure 3 shows the interobserver variability of PMA measurements. The Bland–Altman 95% limits of agreement between the two observers ranged from −219.5 mm^2^ to 218.2 mm^2^ (Figure 3).

As shown in Table 1, 338 patients were divided into three groups based on DXA results: normal (77/338, 22.8%), osteopenia (155/338, 45.9%) and osteoporosis group (106/338, 31.4%). The lowest BMD was 0.916 ± 0.093 g/cm^2^ in the normal group, 0.745 ± 0.063 g/cm^2^ in the osteopenia group, and 0.619 ± 0.088 g/cm^2^ in the osteoporosis group. Age, BMI, and PMA were statistically different in any two groups (*p* < 0.05). Across the normal, osteopenia, and osteoporosis groups, mean age progressively increased, whereas BMI and PMA progressively decreased. In the three groups, the mean age was 62.1 ± 8.8, 68.3 ± 8.2, and 73.8 ± 9.3 years, the mean BMI was 26.7 ± 3.2 kg/m^2^, 24.8 ± 3.6 kg/m^2^, and 23.0 ± 3.4 kg/m^2^, the mean PMA was 24.6 ± 6.0 cm^2^, 21.2 ± 5.8 cm^2^, and 16.4 ± 5.3 cm^2^.

**Table 1:** Patients' characteristics.

**Variables**	**Normal (*n* = 77)**	**Osteopenia (*n* = 155)**	**Osteoporosis (*n* = 106)**	**P1**	**P2**	**P3**	**P4**
Age, yrs	62.1 ± 8.8	68.3 ± 8.2	73.8 ± 9.3	< 0.001	< 0.001	< 0.001	< 0.001
Height, cm	156.9 ± 5.4	154.8 ± 5.4	152.3 ± 6.3	< 0.001	0.029	< 0.001	0.001
Weight, kg	65.7 ± 8.6	59.4 ± 8.8	53.4 ± 8.6	< 0.001	< 0.001	< 0.001	< 0.001
BMI, kg/m^2^	26.7 ± 3.2	24.8 ± 3.6	23.0 ± 3.4	< 0.001	< 0.001	< 0.001	< 0.001
BMD hip, g/cm^2^	0.916 ± 0.094	0.749 ± 0.073	0.627 ± 0.100	< 0.001	< 0.001	< 0.001	< 0.001
BMD L1–L4, g/cm^2^	1.137 ± 0.121	0.962 ± 0.108	0.777 ± 0.107	< 0.001	< 0.001	< 0.001	< 0.001
Lowest BMD, g/cm^2^	0.916 ± 0.093	0.745 ± 0.063	0.619 ± 0.088	< 0.001	< 0.001	< 0.001	< 0.001
PMA, cm^2^	24.6 ± 6.0	21.2 ± 5.8	16.4 ± 5.3	< 0.001	< 0.001	< 0.001	< 0.001

BMI, body mass index; BMD, bone mineral density; PMA, pectoral muscle area; DXA, dual-energy X-ray absorptiometry.

P1, overall comparison among the three groups; P2, normal vs. osteopenia; P3, normal vs. osteoporosis; P4, osteopenia vs. osteoporosis.

### 3.2 Correlation of PMA with BMD

**Figure 4:**
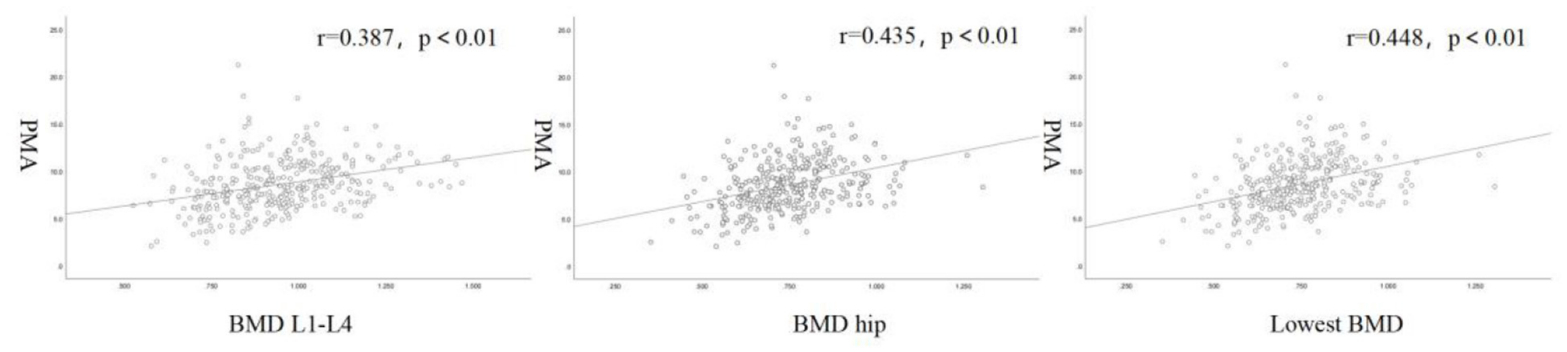
Scatter plots and fitted curves of PMA and BMD.

Table 2 shows that age, BMI and PMA were strongly correlated with BMD. Age was negatively correlated with BMD, and the correlation coefficient with the lowest BMD was 0.505. BMI was positively correlated with BMD, and the correlation coefficient with the lowest bone mineral density was 0.353. PMA was positively correlated with BMD. The correlation coefficients of PMA with lumbar spine and hip BMD were 0.387 and 0.435, respectively. The highest correlation coefficient was found between the PMA and lowest BMD which was 0.448 (Figure 4).

**Table 2:** Correlation of age, BMI, PMA with BMD.

**Variables**	**BMD L1–L4**	**BMD hip**	**Lowest BMD**
Age	−0.358	−0.496	−0.505
BMI	0.432	0.349	0.353
PMA	0.387	0.435	0.448

BMD, bone mineral density; PMA, pectoral muscle area; BMI, body mass index.

### 3.3 Validation of the rationality of linear regression

**Figure 5:**
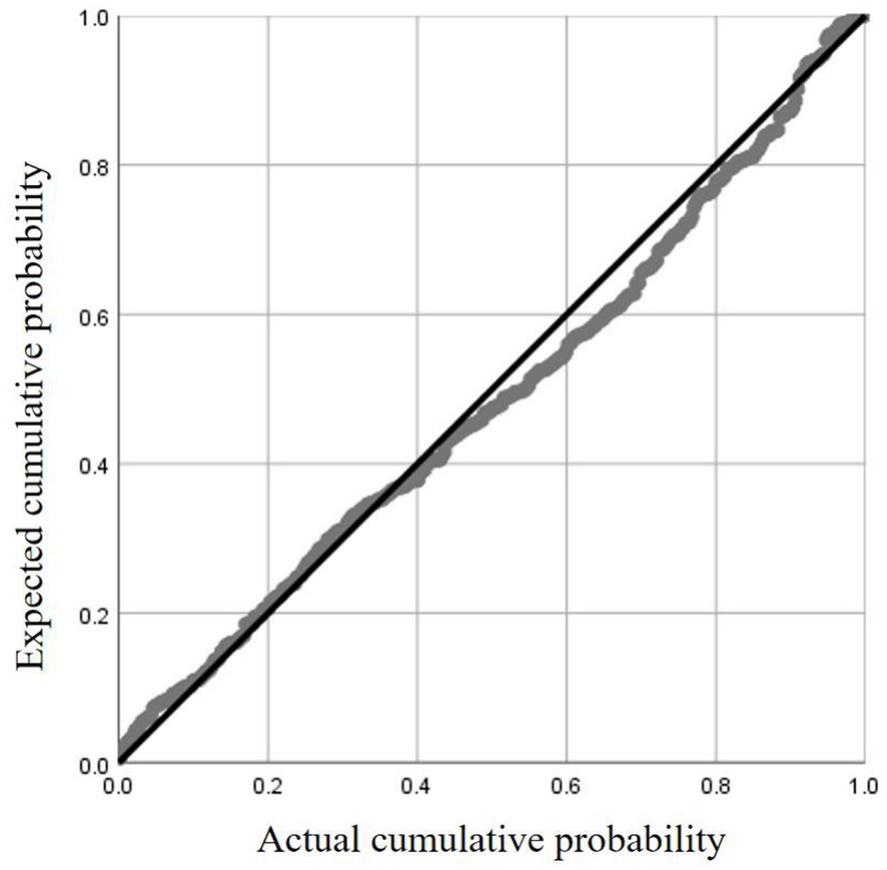
The normal P–P plot of standardized residuals.

**Figure 6:**
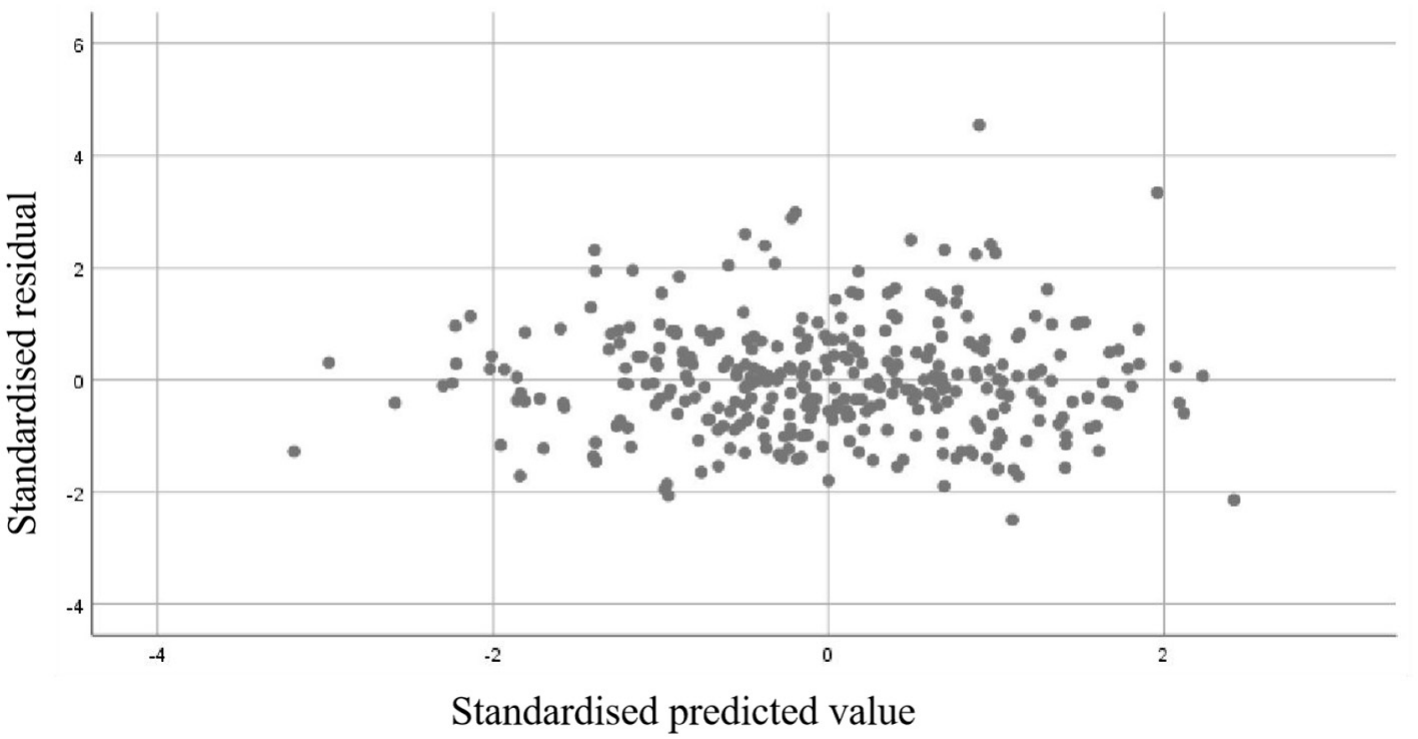
The scatter plot of standardized residuals against predicted values.

To verify the adequacy of the multiple linear regression model constructed with age, BMI, and PMA as predictors of Lowest BMD, the assumptions were examined. The normal P–P plot of standardized residuals showed that the observed cumulative probabilities were closely aligned with the expected diagonal line, indicating that the residuals were approximately normally distributed (Figure 5). Furthermore, the scatter plot of standardized residuals against predicted values exhibited a random distribution around zero, without evident heteroscedasticity or systematic patterns, suggesting that the assumptions of linearity and homoscedasticity were satisfied (Figure 6). These diagnostic results supported the validity of the regression model for subsequent interpretation.

### 3.4 Multiple linear regression analysis

Age, BMI and PMA were found to be linearly associated with Lowest BMD by correlation analysis. We included age, BMI and PMA in multivariate linear regression and tested for multicollinearity between the independent variables by linear regression. Generally, the existence of multicollinearity could be determined if the tolerance was < 0.1 or the variance inflation factor was >10. In this model, the tolerance and VIF for age, BMI, and PMA were 0.852 and 1.174, 0.812 and 1.232, and 0.733 and 1.364, respectively, all within acceptable limits, indicating no substantial multicollinearity. Following the confirmation of regression assumptions and the absence of multicollinearity, the overall significance of the multiple linear regression model was assessed using an F-test. The model was statistically significant (*F* = 61.225, *p* < 0.001) and explained 35% of the variance in the lowest BMD (Adjusted *R*^2^ = 0.35). The linear regression results are shown in Table 3, after adjusting for covariates, Age remained a significant negative predictor of BMD (β = −0.379, *p* < 0.001), while both BMI (β = 0.168, *p* = 0.001) and PMA (β = 0.233, *p* < 0.001) were significant positive predictors. The final formula for the regression model was obtained as: Lowest BMD = 0.858 – 0.005 ^*^ age + 0.006 ^*^ BMI + 0.005 ^*^ PMA. After correcting for age and BMI, independent association between PMA and BMD remained in postmenopausal women. For every 1 cm^2^ increase in PMA, lowest BMD will increase by 0.005 g/cm^2^. By comparing the standardized coefficients, the effect of PMA on lowest BMD is second only to age.

**Table 3:** Multivariable linear regression analysis of independent factors of Lowest BMD.

**Factors**	***B***	**95% CI**	**Standardized β**	***t***	***p***	**Tolerance**	**Variance inflation factor**
Intercept (constant)	0.858	0.724, 0.991	/	12.619	/	/	/
Age	−0.005	−0.007, −0.004	−0.379	−7.964	< 0.001	0.852	1.174
BMI	0.006	0.003, 0.009	0.168	3.436	0.001	0.812	1.232
PMA	0.005	0.003, 0.007	0.233	4.531	< 0.001	0.733	1.364

## 4 Discussion

This study demonstrates that pectoral muscle cross-sectional area on routine chest CT is strongly associated with bone mineral density in postmenopausal patients. Our data suggest that simple area measurements of the pectoralis muscle on routine chest CT can provide useful information about bone health in postmenopausal patients.

CT scans provide a wealth of high-resolution images so that cross-sectional areas of tissue can be obtained (17). Among them, routine CT scanning of the chest is widely used for routine physical examination of the population and diagnosis of lung diseases, that is utilized much more frequently than other parts of the body (13). It has been demonstrated that skeletal muscle measurements at the level of the T4 vertebrae in chest CT are as good as those in the abdomen for assessing the patient's whole body muscle level. It has been shown that PMA from individual axial CT slices is strongly correlated with pectoral muscle volume (*r* = 0.89–0.91, *p* < 0.001) (18). The measurement of PMA at the T4 level has been proposed as a surrogate marker for sarcopenia (12). Therefore, in this study, the PMA at the T4 level was used as a measurement target. The measurements of the two workers in this study were in good agreement and had a small range of fluctuation, similar to the results of other studies (18). Our findings are in line with previous studies reporting a close association between skeletal muscle mass and BMD. Kuriyama et al. (7) reported that thigh muscle cross-sectional area was significantly correlated with BMD and osteoporosis status in middle-aged and older adults, supporting the concept of muscle–bone coupling. Similarly, Ekin and Huang demonstrated that reduced paraspinal muscle area on CT was associated with lower bone density in geriatric populations (19, 20).

Compared to studies primarily focusing on abdominal, paraspinal, or thigh muscles, this research assessed pectoral muscle area at the T4 vertebral level using conventional chest CT scans—the most frequently performed CT examination. We observed a correlation between PMA and lowest BMD (*r* = 0.448) comparable to correlations reported for other muscle sites, suggesting pectoral muscle measurement may reflect systemic bone mass status (7, 21). Notably, unlike previous studies that included mixed-gender or male-dominated cohorts, this research specifically targeted postmenopausal women—a population at extremely high risk for both osteoporosis and sarcopenia.

Estrogen is an important hormone that regulates the metabolism and function of skeletal muscle and bone, either directly or indirectly through the estrogen receptor (6). Postmenopausal women experience a decline in estrogen, which disrupts bone remodeling by altering the balance between osteoblast-mediated bone formation and osteoclast-mediated bone resorption, leading to bone loss, and also impairs skeletal muscle maintenance by affecting muscle stem cell proliferation and protein homeostasis (22–26). Because of this, women over the age of fifty are more likely to suffer from osteoporosis and sarcopenia than men (27). Therefore, postmenopausal women were selected for this study. Data show that in the postmenopausal female population, from the normal, osteopenia to the osteoporosis group, the average age gradually increases while the BMI gradually decreases. Age is the main risk factor for osteoporosis. As a person ages, body functions gradually deteriorate, including loss of bone mass and muscle (28). BMI reflects the degree of body fatness and thinness. The incidence of osteoporosis is higher in thin populations which may be related to the effect of mechanical loading on bone mass. Appropriate weight-bearing exercise has been demonstrated to increase bone mass in patients (29, 30).

The study showed that there was no multicollinearity between age, BMI and PMA. Even after correcting for age and BMI, PMA remains a positive independent predictor of BMD. In addition to the effects of estrogen on bone, the association between bone and muscle cannot be ignored in postmenopausal women. Skeletal muscle is interconnected with bone. Mechanotransduction signals lead to the production of biochemical factors called osteokines and myokines in bone and skeletal muscle, respectively, bridging the gap between communication between bone and muscle (31, 32). Collectively, myokines and osteokines mediate bidirectional communication between muscle and bone, thereby regulating both bone mineral density and muscle mass through coordinated effects on cellular metabolism and protein homeostasis (33–36).

## 5 Strengths and limitations

To our knowledge, this study represents the first investigation into the association between PMA derived from chest CT and BMD in postmenopausal women in China. Several limitations of this study should be acknowledged. First, this was a single-center, retrospective study, which may limit the generalizability of our findings. Second, factors such as smoking history, physical activity, and nutritional status, which may affect muscle and bone mass, were not accounted for. Future research should include multi-center, prospective studies with larger sample sizes and diverse populations. Additionally, incorporating lifestyle factors such as physical activity, smoking, and diet could clarify their influence on the muscle-bone interaction. Finally, the development of automated methods for opportunistic PMA assessment on routine chest CT may facilitate large-scale osteoporosis screening in clinical practice.

## 6 Conclusion

In conclusion, there is a strong correlation between PMA and BMD in postmenopausal women. A reduction in muscle mass indicates the presence of bone loss in patients. PMA assessment on routinely acquired chest CT images may serve as a complementary and opportunistic tool to identify individuals at increased risk of low bone mass, particularly in clinical settings where DXA is not readily available. This offers a new approach for opportunistic screening for osteoporosis.

## Data Availability

The original contributions presented in the study are included in the article/supplementary material, further inquiries can be directed to the corresponding author.
